# Lactoferrin Decreases the Intestinal Inflammation Triggered by a Soybean Meal-Based Diet in Zebrafish

**DOI:** 10.1155/2016/1639720

**Published:** 2016-05-10

**Authors:** Pilar E. Ulloa, Camila J. Solís, Javiera F. De la Paz, Trevor G. S. Alaurent, Mario Caruffo, Adrián J. Hernández, Patricio Dantagnan, Carmen G. Feijóo

**Affiliations:** ^1^Departamento de Ciencias Biologicas, Facultad de Ciencias Biologicas, Universidad Andrés Bello, Republica 217, 8370146 Santiago, Chile; ^2^Núcleo de Investigación en Producción Alimentaria, Escuela de Acuicultura, Facultad de Recursos Naturales, Universidad Católica de Temuco, Avenida Rudecindo Ortega 02950, Casilla 15D, 4780000 Temuco, Chile; ^3^Interdisciplinary Center for Aquaculture Research, 4070007 Concepción, Chile

## Abstract

Intestinal inflammation is a harmful condition in fish that can be triggered by the ingestion of soybean meal. Due to the positive costs-benefits ratio of including soybean meal in farmed fish diets, identifying additives with intestinal anti-inflammatory effects could contribute to solving the issues caused by this plant protein. This study evaluated the effect of incorporating lactoferrin (LF) into a soybean meal-based diet on intestinal inflammation in zebrafish. Larvae were fed with diets containing 50% soybean meal (50SBM) or 50SBM supplemented with LF to 0.5, 1, 1.5 g/kg (50SBM+LF0.5; 50SBM+LF1.0; 50SBM+LF1.5). The 50SBM+LF1.5 diet was the most efficient and larvae had a reduced number of neutrophils in the intestine compared with 50SBM larvae and an indistinguishable number compared with control larvae. Likewise, the transcription of genes involved in neutrophil migration and intestinal mucosal barrier functions (*mmp9*,* muc2.2*, and *β-def-1*) were increased in 50SBM larvae but were normally expressed in 50SBM+LF1.5 larvae. To determine the influence of intestinal inflammation on the general immune response, larvae were challenged with* Edwardsiella tarda*. Larvae with intestinal inflammation had increased mortality rate compared to control larvae. Importantly, 50SBM+LF1.5 larvae had a mortality rate lower than control larvae. These results demonstrate that LF displays a dual effect in zebrafish, acting as an intestinal anti-inflammatory agent and improving performance against bacterial infection.

## 1. Introduction

Intestinal inflammation in fish is a detrimental condition that affects growth and the ability to respond to pathogens [1]. Preventing this pathology is of particular relevance for fish farming as small fish size and/or high fish mortality drastically affect the competitiveness and profitability of the aquaculture industry.

Most commercially important fish are carnivorous and require a high-protein diet, usually provided through fishmeal [2]. However, increased aquaculture production has limited fishmeal availability, leading to the use of plant proteins in fish diets [2]. Soybean meal, which is widely available and economical, has a balanced amino acid profile, and contains a high amount of digestible proteins, is currently the most common plant protein source used in fish feed [3]. Studies in different fish species, such as Atlantic salmon (*Salmo salar*) [4], rainbow trout (*Oncorhynchus mykiss*) [5], carp (*Cyprinus carpio L.*) [6], Nile tilapia (*Oreochromis niloticus*) [7], gilthead seabream (*Sparus aurata*) [8], and zebrafish (*Danio rerio*) [9, 10], have shown that the inclusion of soybean meal in the diet triggers intestinal inflammation [11–14]. Nevertheless, the advantages of soybean meal outweigh its disadvantageous effects to fish intestines.

To optimize the use of this plant protein, there is an ongoing search to find dietary additives that could protect the intestine from the effects of soybean meal. Traditionally, additives have been incorporated into fish diets to control diseases, increase health, and improve the stress response [1, 15–19]. However, little focus has been given to the use of additives for controlling intestinal inflammation. At present, there are only two studies that evaluate the effects of additives, specifically mannan-oligosaccharide and *β*-glucans, on soybean meal-triggered intestinal inflammation in farmed fish [1, 17]. These investigations indicate that only mannan-oligosaccharide is able to decrease, to varying degrees, the altered intestinal histology observed in fish fed with diets including low amounts of soybean meal [1, 17].

Lactoferrin (LF) is an abundant iron-binding glycoprotein secreted by epithelial cells and contributes to the composition of bodily fluids in mammals, such as milk, tears, saliva, bile, and pancreatic fluid [20, 21]. Specific LF receptors are present on the surface of different tissues and cell types, including the gastrointestinal tract, lungs, neutrophils, and eosinophils [22, 23]. Previous research has demonstrated that this glycoprotein has antimicrobial activity and can stimulate cytokine production, enhance cell proliferation, and regulate mucosal immunity [20, 24–26]. Due to these properties, LF has been used in prophylactic treatments for fish against different infectious diseases [25, 27–29]. Furthermore, LF exerts a potent anti-inflammatory effect in different tissues, mainly by reducing immune cell recruitment to inflammatory sites. During influenza virus infection, LF reduces the number of infiltrating leukocytes in bronchoalveolar lavage fluid in humans [30]. Likewise, LF can reduce eosinophil infiltration to the pigs small intestine in a mechanism independent of cytokine [31, 32]. Moreover, an* in vivo* study in rats and mice demonstrated that orally administered LF prevents intestinal injury triggered by nonsteroidal anti-inflammatory drugs. The authors related this effect to the attenuation of neutrophil migration to the intestine [33]. Despite these various related studies, there are currently no reports on the possible role of LF as an intestinal protector in fish and less so on the possible protective effects of LF against soybean meal-induced intestinal inflammation in fish.

Factors contributing to this lack of information are the high costs and long-term assays involved in evaluating the use of additives in the aquaculture industry, which is in addition to the challenge of working with minimal precedents on the cellular and molecular processes in many farmed fish species. Therefore, an alternative research strategy is to perform preliminary studies in a model fish in which many diets can be assessed in a short period and at low costs. Moreover, the selected fish model should facilitate understanding the biological processes triggered by different diets. Considering the extensive biological literature and powerful biotechnological tools available for zebrafish (*D. rerio*), this teleost fish is an ideal organism for immune-nutrition research [34].

One key advantage of zebrafish is the availability of transgenic lines with certain fluorescently-labeled innate immune cells, such as neutrophils [35]. Since the hallmark of inflammation is neutrophil migration, these cells can be used as inflammatory markers. This strategy has been used before by Hedrera et al. [9], who demonstrated that soybean meal consumption by zebrafish results in inflammatory side effects similar to those observed in commercially farmed fish. A primary advantage of using transgenic, fluorescently labeled zebrafish is that the inflammatory process can be very quickly observed, even before histological effects become recognizable.

This study evaluated the effects of LF on intestinal inflammation induced by a soybean meal-based diet in zebrafish. By using the Tg(BACmpo:GFP)^i114^ transgenic zebrafish line, neutrophil migration as part of the intestinal inflammatory process was monitored* in vivo*. To complement this data, the transcriptional levels of different markers related to mucosal barrier functions as matrix metallopeptidase 9, mucin 2.2, and beta-defensin 1 (*mmp9*,* muc2.2*, and *β-def-1*) and lipid absorption like the fatty-acid-binding proteins 2 and 6 (*fabp2* and* fabp6*) were evaluated. Finally, the influence of intestinal inflammation on the immune response to* Edwardsiella tarda* infection was assessed.

## 2. Material and Methods

### 2.1. Zebrafish Strains and Maintenance

Zebrafish were maintained and raised at the Laboratory of Developmental Biology, Universidad Andrés Bello, Chile, according to standard protocols [37]. The Tg(BACmpo:GFP)^i114^ transgenic zebrafish line was used [35]. All embryos were collected through natural spawning according to Kimmel et al. [38]. Eggs were incubated in petri dishes at 28°C for three days in the E3 medium (5 mM NaCl, 0.17 mM KCl, 0.33 mM CaCl_2_, and 0.33 mM MgSO_4_, with methylene blue, equilibrated to pH 7.0) [39]. Embryonic and larval stages are expressed in hours postfertilization (hpf) or days postfertilization (dpf). All animal-handling procedures were approved by the Committee of Animal Bioethics of the Universidad Andrés Bello.

### 2.2. Experimental Diets

Five diets were formulated and prepared (Table 1). The positive control diet contained 50% soybean meal (50SBM), while the negative control diet contained fishmeal as the primary protein source (100FM) [9]. Additionally, a diet normally used for zebrafish larvae (ZFP, sera Micron®, Heinsberg, Germany) was used as a second negative control. To evaluate the intestinal protective effect of LF, three batches of the 50SBM diet were supplemented with bovine LF obtained from milk (Lactoferrin 100% S60, Natural Healthy Concepts, Appleton, WI, USA) in concentrations of 0.5, 1.0, or 1.5 g/kg (50SBM+LF0.5; 50SBM+LF1.0; and 50SBM+LF1.5, resp.) [40]. Likewise, one batch of the 100FM diet was supplemented with 1.5 g/kg of bovine LF (100FM+LF1.5). All diets were supplemented with a standard vitamin and mineral premix and formulated to be isoenergetic, isonitrogenous, and isolipidic. Each feed diet was prepared by cooking-extrusion in a twin screw extruder (CLEXTRAL BC-21, Clextral, Firminy, France) with a 2 mm diameter. The resulting moist pellets were oven-dried at 60°C for approximately eight hours and then coated with fish oil, according to the formulation for each diet, using a laboratory vacuum coater (Dinnissen Model VC10, Sevenum, Netherlands). The pellets were subsequently crumbled, screened to the appropriate particle size (75 *μ*m), and stored at −20°C until use in the feeding trials.

### 2.3. Experimental Feeding Period

Experimental feeding was performed as previously described by Hedrera et al. [9]. Briefly, 45 larvae were fed two times daily from 5 to 8 dpf. The last feed was given at least 14 h before larval fixing to promote intestine emptying.

### 2.4. Immunohistochemistry and Sudan Black B Staining

Immunohistochemistry was performed as previously described by Feijoo et al. [41]. The following antibodies were used: rabbit anti-Green Fluorescent Protein (anti-GFP) (Cat. number A11122, Invitrogen, Carlsbad, CA, USA) and anti-rabbit peroxidase (Cat. number A8275, Sigma, St. Louis, MO, USA). Additionally, Sudan Black B staining was performed following the manufacturer's protocol (Cat. number 3801, Sigma-Aldrich, St. Louis, MO, USA). The neutrophils present in the intestine were quantified within a defined area that included the mid and posterior intestine. At least 28 larvae were analyzed per diet in three independent experiments.

### 2.5. Quantitative Polymerase Chain Reaction (qPCR)

Larvae fed with the control and experimental diets were sampled at the end of each treatment for total RNA extraction. Total RNA was extracted from a pool of ~60 larval intestines per diet. The whole intestine was dissected from larvae anesthetized in tricaine methanesulfonate using sterile instruments. Samples were stored in the RNAlater solution and then homogenized in the TRIzol Reagent (Cat. number 15596-026, Invitrogen, Carlsbad, CA, USA) according to the manufacturer's instructions. The corresponding cDNA were synthesized from 2.5 *μ*g of total RNA using SuperScript II Reverse Transcriptase (Cat. number 100004925, Invitrogen, Carlsbad, CA, USA) and Oligo-dt primers. Primer sequences and the efficiencies are shown in Table 2. qPCR was performed with the ABI 7300 Real-Time PCR system using the Maxim SYBR Green/ROX qPCR Master Mix (2x) (Fermentas, Waltham, MA, USA) following the manufacturer's instructions. A 15 *μ*L reaction volume was used, containing 1 *μ*L of 10-fold diluted cDNA. The PCR was run with a ten-minute activation and denaturation step at 95°C, followed by 40 cycles of 30 s at 95°C, 30 s at 57–60°C, and 30 s at 72°C. Reaction specificity was verified using melting curve analysis and the absence of primer dimmers. Standard curves were obtained for each pair of primers by plotting Ct values against the log_10_ of five different dilutions of a cDNA mix solution for all analyzed samples. Real-time PCR efficiency (*E*) was calculated from a standard curve according to the equation *E* = 10(−1/slope) [42]. Relative expression was calculated with the Pfaffl method [42], and the 50SBM diet was used as a calibrator.

### 2.6. cDNA Cloning, Probe Synthesis, and Whole-Mount* In Situ* Hybridization

The* fabp2* gene was cloned using the following primers: F-5′-CGACCGCAATGAGAACTACGAGAA-3′ and R-5′-CTCACAGGTGCAAATGACACGA-3′ (gene ID: NM_131431.1) from a 9 dpf cDNA library. A 529 base pair cDNA fragment was cloned into the pGEM-T easy vector (Promega, Madison, WI, USA), which was digested with the* ApaI* restriction enzyme. The anti-sense riboprobe was then synthesized using the* Sp6* RNA polymerase. The* fabp6* clone was kindly provided by Oehlers et al. [43].* In situ* hybridization was performed as previously described by Jowett and Lettice [44].

### 2.7. *Edwardsiella tarda* Challenge

*Edwardsiella tarda* FL60 was kindly provided by Dr. Phillip Klesius (USDA, Agricultural Research Service, Aquatic Animal Health Research Unit). The* E. tarda* culture was grown as previously described by Harvie et al. [45] and Van Soest et al. [46], with some modifications. Briefly,* E. tarda* was grown overnight at 28°C in a trypticase soy broth medium with agitation. The overnight culture was diluted to 1 : 100 in fresh trypticase soy broth medium and incubated at 28°C to reach 10^8^ CFU/mL. The culture was pelleted by centrifugation at 1500 g for five minutes, washed with water from the aquarium, and repelleted to recover the bacteria. The clean* E. tarda* was suspended in water from the aquarium to reach 10^8^ CFU/mL. After four days of feeding, a group of 30 larvae were challenged for 5 h in 200 mL of the* E. tarda* water media and were subsequently transferred to a tank with new, clean water. Each respective diet was resumed for the remainder of the trial period, and larvae mortality was monitored every 12 h for four days.

### 2.8. Statistical Analysis and Imaging

The data were analyzed using a nonparametric, Kruskal-Wallis, one-way ANOVA, and Dunn multiple comparisons test. The data were normally distributed (the D'Agostino and Pearson normality test), but variance was not homogenous (the Brown-Forsythe test). Survival data were analyzed using Kaplan-Meier and group differences were analyzed by the log-rank test, using the Bonferroni correction for multiple comparisons. All analyses were performed using Prism 4 (GraphPad Software, La Jolla, CA, USA). Significance was established for all analyses at *P* < 0.05. Lateral view photographs of larvae were taken in an Olympus SZX16 stereoscope with a QImaging MicroPublisher 5.0 RVT camera. Images were processed with Photoshop CS4 or ImageJ v1.44.

## 3. Results

### 3.1. Effect of Soybean Meal Diet Supplemented with Lactoferrin on Intestinal Inflammation

**Figure 1:**
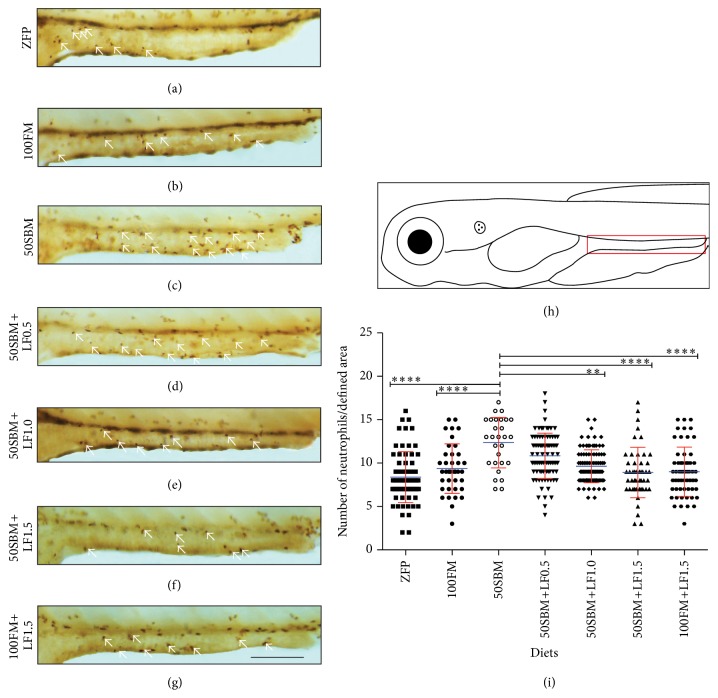
Effect of different lactoferrin doses on neutrophil migration to the intestine. (a–g) Lateral view of mid and posterior intestine of Tg(BACmpo:GFP)^i114^ larvae of 9 dpf after four days of feeding with different diets (ZFP, 100FM, 50SBM, 50SBM+LF0.5, 50SBM+LF1.0, 50SBM+LF1.5, and 100FM+LF1.5). Neutrophils were quantified through immunohistochemistry against GFP (white arrows). (h) Larva scheme with the intestinal region of interest demarcated with a red rectangle. (i) The experiments were conducted with at least 28 larvae per treatment in three different assays. Statistical analysis was performed by comparing data sets with the 50SBM diet through one-way ANOVA. The graph is a representation of three different results. ^*∗∗*^ *P* < 0.01; ^*∗∗∗∗*^ *P* < 0.0001. Bar scale = 200 *μ*m.

To determine if LF exerted an intestinal protector effect, by preventing or decreasing inflammation, this additive was incorporated to the soybean meal-based diet (50SBM), thereby generating three experimental diets (50SBM+LF0.5, 50SBM+LF1.0, and 50SBM+LF1.5). The 50SBM diet was used as a positive control that triggers inflammation, while the 100FM and ZFP diets were used as negative controls [9]. To determine the extent of intestinal inflammation, the amount of neutrophils present in the intestine was quantified (Figure 1).

Confirming previously published data, the results indicated a clear increase in the number of neutrophils situated in the intestine of larvae fed with a 50SBM diet compared to larvae fed with the ZFP and 100FM diets (Figures 1(a)–1(c) and 1(i)). In larvae fed with the 50SBM+LF0.5 diet, the number of neutrophils located in the intestine showed no significant differences compared with 50SBM larvae (Figures 1(d), 1(c), and 1(i)). On the other hand, larvae fed with the 50SBM+LF1.0 and 50SBM+LF1.5 diets presented a decreased number of neutrophils in the intestine compared to the 50SBM group and, more importantly, these larvae were indistinguishable from those fed the 100FM and ZFP diets (Figures 1(e), 1(f), and 1(i)). Of significance, the amount of intestine-located neutrophils in larvae fed the 50SBM+LF1.5 diet was similar to larvae fed the 100FM+LF1.5 diet (Figures 1(f), 1(g), and 1(i)).

To corroborate immunohistochemistry data, Sudan Black B staining was performed to specifically label leukocytes. Total concordance was found between the results obtained with the two techniques (Supplementary Figure 1, in Supplementary Material available online at http://dx.doi.org/10.1155/2016/1639720).

Since LF concentrations of 1.0 and 1.5 g/kg exerted similar effects, subsequent analyses were performed using the 50SBM diet supplemented by 1.5 g/kg of LF (i.e., 50SBM+LF1.5).

### 3.2. Effect of Soybean Meal Diet Supplemented with Lactoferrin on Genes Related to Intestinal Mucosal Function

**Figure 2:**
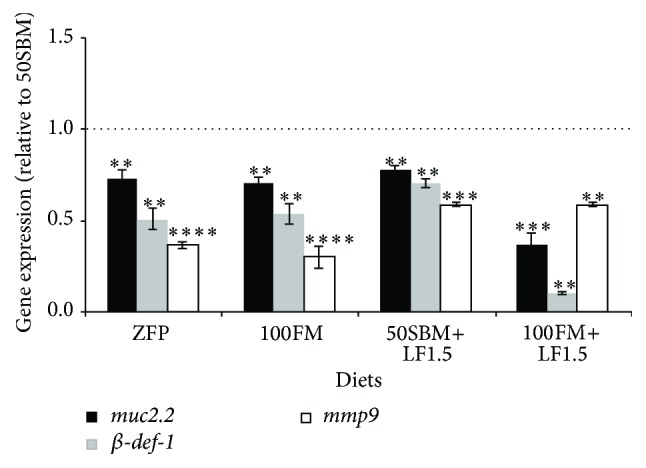
Effect of lactoferrin on transcriptional levels of mucosal barrier functional markers. Transcription levels of* muc2.2*, *β-def-1*, and* mmp9* were quantified by qPCR. Quantification was performed from a pool of ~60 intestines of larvae of 9 dpf after four days of feeding with different diets (ZFP, 100FM, 50SBM, 50SBM+LF1.5, and 100FM+LF1.5). All data were normalized with *β-actin* and* rpl13α*. The data from the different diets were compared to the 50SBM diet (dotted lines). ^*∗∗*^ *P* < 0.01; ^*∗∗∗*^ *P* < 0.001; ^*∗∗∗∗*^ *P* < 0.0001.

To evaluate the effect of LF on genes involved in intestinal mucosal function, the transcripts of* muc2.2*, *β-def-1*, and* mmp9* were determined through qPCR. The relative expressions of these genes in response to the different diets were compared against expressions in larvae fed with the 50SBM diet (Figure 2, dotted line). As expected, in larvae fed with the ZFP and 100FM control diets, the transcriptional levels of* muc2.2*, *β-def-1* and* mmp9* were significantly lower than in larvae fed with the 50SBM diet (*P* < 0.001). This same result was observed in larvae fed the 100FM+LF1.5 diet. Notably, the mRNA levels in larvae fed with the 50SBM+LF1.5 diet were comparable to those observed in the ZFP and 100FM groups and were significantly lower than those in larvae fed with the 50SBM diet (*P* < 0.001).

### 3.3. Effect of Soybean Meal Diets Supplemented with Lactoferrin on Genes Related to Intestinal Lipid Absorption

**Figure 3:**
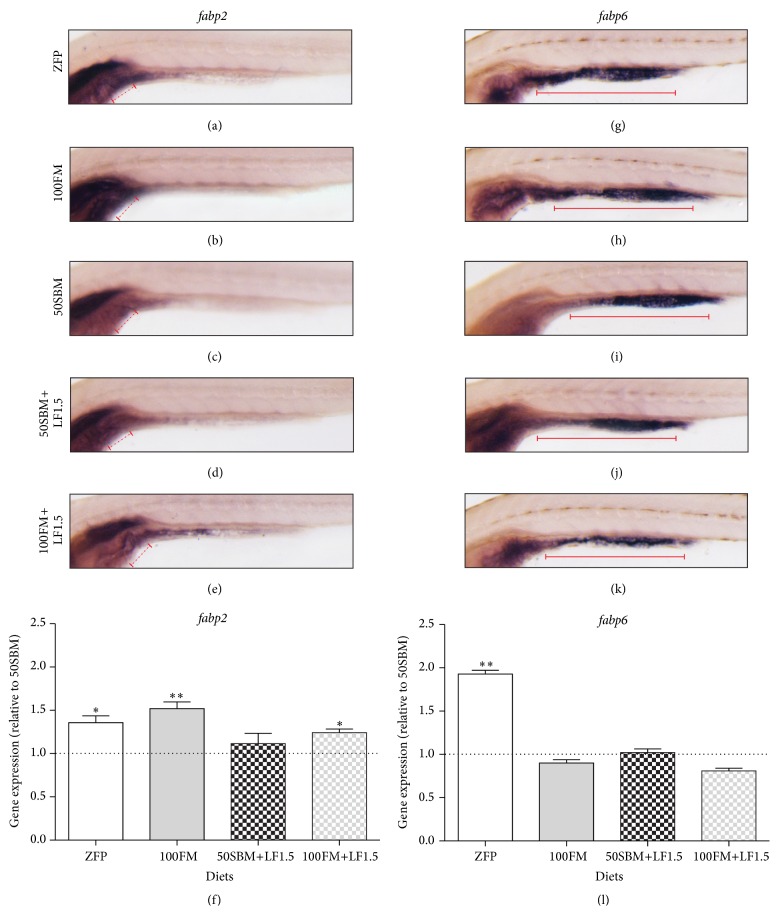
Effect of lactoferrin on lipid absorption markers. (a–e)* fabp2* and (g–k)* fabp6* mRNA expression pattern was analyzed by whole-mount* in situ* hybridization. Lateral view of larvae of 9 dpf after four days of feeding with different diets (ZFP, 100FM, 50SBM, 50SBM+LF1.5, and 100FM+LF1.5). (a–e)* fabp2* expression was restricted to anterior intestine (red dotted line). (g–k)* fabp6* expression was observed in the whole intestine, with a stronger expression in the mid and posterior gut (red continued line). (f and l) The transcriptional levels of* fabp2* and* fabp6* were quantified by qPCR. Data were normalized with *β-actin* and* rpl13α* and compared to 50SBM diet (dotted line). ^*∗*^ *P* < 0.05; ^*∗∗*^ *P* < 0.01.

To evaluate the effect of a 50SBM diet and LF supplementation on the expression of genes related to the lipid absorption process in the intestine,* in situ* hybridization and qPCR were performed for the markers* fabp2* and* fabp6* at 9 dpf. The* fabp2* expression was restricted to the anterior intestine, with a reduction towards the mid and posterior intestine (Figures 3(a)–3(e), red dotted line). In contrast,* fabp6* was expressed at the mid and posterior intestine (Figures 3(g)–3(k)). The expression of* fabp6* in the defined area was similar between larvae fed with the different diets (Figures 3(g)–3(k), red continued line). Likewise, qPCR analysis revealed a significant upregulation in the expression of* fabp2* in control diets (ZFP, 100FM, and 100FM+LF1.5) in comparison to those fed with 50SBM (Figure 3(f)). The transcriptional expression of* fabp6* between control and experimental larvae did not vary, except in larvae fed the ZFP diet, where significant upregulation was observed (*P* < 0.01) (Figure 3(l)).

### 3.4. Effect of Intestinal Inflammation on Immune Performance against* Edwardsiella tarda*

**Figure 4:**
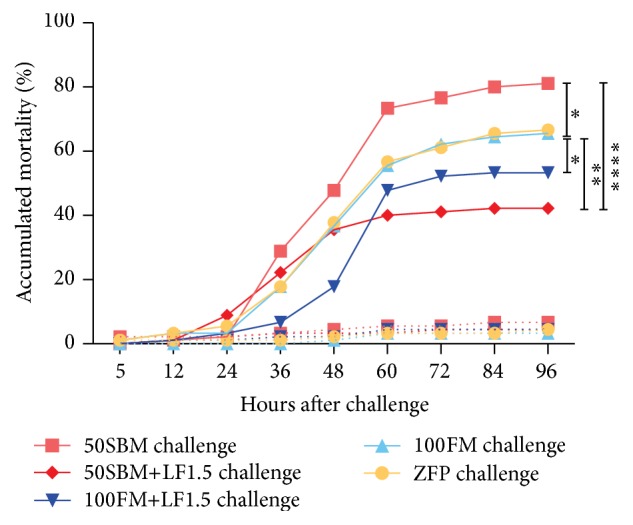
Effect of lactoferrin on fish mortality after pathogen challenge. Tg(BACmpo:GFP)^i114^ larvae were challenged with* Edwardsiella tarda* after four days of feeding with different diets at 9 dpf (ZFP, 100FM, 50SBM, 50SBM+LF1.5, and 100FM+LF1.5). Mortality was monitored immediately after the challenge and every 12 h over four days until 13 dpf. Statistical analysis was performed using survival curve analysis with the log-rank test against the 100FM and 50SBM diets. ^*∗*^ *P* < 0.05; ^*∗∗*^ *P* < 0.01; ^*∗∗∗∗*^ *P* < 0.0001. Continuous lines represent challenged larvae while dotted lines represent control larvae.

To determine if intestinal inflammation affected the immune performance of the different larvae groups, a challenge assay was performed using the enterobacteria* E. tarda* (Figure 4). The challenged larvae fed with the 50SBM diet presented significantly higher mortality rates than those fed with the 100FM or ZFP diets (*P* < 0.01). However, larvae fed the 50SBM+LF1.5 diet showed a drastic decrease in accumulated mortality at the end of the experiment compared to 50SBM (*P* < 0.01), and mortality rates were even lower in the 50SBM+LF1.5 group than in larvae fed with the 100FM or ZFP diets. Similarly, larvae fed with the 100FM+LF1.5 diet also showed reduced accumulated mortality compared to the 100FM diet (*P* < 0.01). The mortality of larvae fed with the 50SBM+LF1.5 or 100FM+LF1.5 diets was comparable, and no significant differences existed between these groups.

## 4. Discussion

This is the first study to provide evidence that orally administered LF protects the fish intestine from the inflammatory effect induced by soybean meal. This observation widens the opportunity for using this plant protein in the fish nutrition industry.

The obtained results suggest that LF reduces neutrophil recruitment to the intestine and that this effect is dose-dependent. In line with this attenuated neutrophil migration, there was a downregulation in the transcription of* mmp9*, an enzyme that degrades the basement membrane to facilitate cell migration and infiltration to affected tissue [47]. Moreover, neutrophils are the major contributors of MMP-9 during intestinal inflammation in mammals [48, 49].

The effect of LF as an intestinal protector has been previously reported in rodents. Dial et al. [33] found that LF protects the intestine of rats and mice from the effects of nonsteroidal anti-inflammatory drugs. The authors speculated that LF could modulate neutrophil function, attenuating neutrophil migration to the intestine. However, the mechanism by which LF inhibits leukocyte migration is still unknown. There is evidence that this protein reduces the integrin-dependent adherence of eosinophils [50] and inhibits the expression of adhesion molecules, such as E-selectin and ICAM-1, in the vascular endothelium [51]. Considering that the presence of these adhesion molecules on the surface of the endothelium is a key step during leukocyte recruitment to affected sites, the absence or low levels of adhesion molecules could possibly explain the effect observed in the present study on neutrophil migration. On the other hand, LF can also regulate cytokine production in mice. Therefore, another possible scenario is that, by modulating cytokine levels, LF decreases neutrophil migration to the intestine. Data supporting this hypothesis indicate that LF can suppress the proinflammatory cytokines tumor necrosis factor alfa [52] and IL-1 [53], in addition to promoting the anti-inflammatory cytokine IL-18 in the gut [54]. Moreover, it is possible that the decreased neutrophil migration triggered by LF is partly a consequence of the inhibition of different proinflammatory cytokines.

Regarding the effect of intestinal inflammation on survival rate, the challenge assay with* E. tarda* results clearly indicated that an inflammatory process significantly affects the immune response against pathogens. The present results showed that the survival rate of larvae fed with soybean meal was almost half of that observed in larvae fed with fishmeal (19% and 35%, resp.). Similar results have been reported in adult zebrafish specimens. Specifically, oxazolone-induced intestinal inflammation resulted in treated zebrafish being more susceptible to* E. tarda* infection than healthy zebrafish [55]. Therefore, it is not the soybean meal* per se* that affects the immune response against pathogens, but rather intestinal inflammation. Moreover, in a mouse colitis model with concomitant intestinal inflammation, infection with* Salmonella enterica* was facilitated [56].

The present results further suggest that the effect of LF on the intestine is not limited to preventing intestinal inflammation but that LF can also influence larvae performance against pathogen. This was evidenced in the challenge assay when comparing the survival rates of larvae fed diets with or without LF. In the case of larvae fed with fishmeal that did not have intestinal inflammation, the survival rate increased from 35% to 47% when LF was incorporated. Interestingly, transcriptional analyses of* muc2.2* and *β-def-1* in larvae fed with LF were indistinguishable from control larvae. These results indicate that LF did not improve the mucosal barrier function of the host immune response. Defensins, including *β-def-1*, are crucial antimicrobial peptides that protect the intestine against infection as a result of antibacterial and immunomodulatory properties [55, 57, 58]. Likewise, mucins such as* muc2.2* form part of the mucosal defense system present in the mucous gel layer that covers the luminal surface of the gastrointestinal tract. This viscoelastic protective barrier forms the first line of defense to the external environment [59]. Additionally, in a chemically induced zebrafish model of intestinal inflammation, the mucus layer increases [60].

Therefore, the presently observed increase in survival rate could be the result of LF directly acting against the invading bacteria. Indeed, LF has an antimicrobial effect against a broad spectrum of bacteria, mainly Gram-negative bacteria present in the gut [61]. By inhibiting the overgrowth and/or colonization of bacterial pathogens, LF could promote a healthy condition. This inhibitory action of LF could be achieved by the following three events: (1) chelating ferric iron necessary for bacterial growth; (2) destabilizing microbial membranes; or (3) modifying microbial adherence to host cells independent of the microbe's iron-binding properties [62]. Obviously, the antimicrobial activities of LF are not absolute and permit the growth of commensal bacteria [63].

## 5. Conclusions

The present study provides novel and relevant data regarding the effects of LF on fish physiology, especially in relation to intestinal inflammation. In light of the obtained results, the supplementation of fish diets with LF appears to be a plausible alternative to cope, at least in part, with two major problems currently affecting the aquaculture industry-soybean meal-triggered enteritis and pathogenic infections.

## Supplementary Material

The supplementary Figure 1 shows the Sudan Black B staining in order to label leukocytes in the intestine of 9dpf larvae fed with the different diets. Total concordance between these result and those obtained from the immunohistochemistry analysis were found.

## Figures and Tables

**Table 1:** Ingredients and nutrient composition of experimental diets.

	100FM	50SBM	Different doses of LF incorporated into diets			
			50SBM+LF0.5	50SBM+LF1.0	50SBM+LF1.5	FM+LF1.5
*Ingredients g/kg*						
Fishmeal	610	250	250	250	250	610
Soybean meal	0	500	500	500	500	0
Wheat grain meal	255	115	115	115	115	255
Starch	45	45	45	45	45	45
Fish oil	30	60	59.5	59	58.5	28.5
Vitamineral mix^1^	30	30	30	30	30	30
Cellulose	30	0	0	0	0	30
Lactoferrin	0	0	0.5	1	1.5	1.5
Total	1000	1000	1000	1000	1000	1000

*Analytical composition (dry base, %)*						
Dry matter	95.3	93.5	94.0	94.0	93.04	94.27
Total proteins	46.4	43.5	43.4	43.4	43.46	44.40
Total lipids	7.8	8.4	7.6	7.6	6.78	6.39
Ash	12.6	9.7	9.8	9.8	8.25	9.76
Gross energy (MJ/kg)	20.0	20.3	20.2	20.2	20.2	20.0

^1^As recommended by the National Research Council, 1993 [36].

**Table 2:** Primer sequences used for amplification of specific genes through RT-qPCR.

Gene	Forward primer	Reverse primer	Amplicon (bp)	Gene ID	Efficiency
*muc2.2*	ACACGCTCAAGTAATCGCACAGTC	TCAGCGAGTGTTTGGCTCACTT	137	XM_002667543	1.82
*mmp9*	CATTAAAGATGCCCTGATGTATCCC	AGTGGTGGTCCGTGGTTGAG	142	NM_213123.1	1.89
*β-def-1*	CTCCTTGTCGTACTAGCATTGCAC	ACACACTCCTTGTCTGCAAACACC	99	NM_001081553.1	1.86
*fabp2*	TCAACGGGACCTGGAAAGTC	CCCATTTGTTCCATGAACTTCTC	61	NM_131431.1	1.86
*fabp6*	CTCCGCTCAATCAACACCAA	TGAGATTCGGTTTCCCACTTG	59	NM_001002076.1	1.93
*β-actin*	TTCTGGTCGTACTACTGGTATTGTG	ATCTTCATCAGGTAGTCTGTCAGGT	144	NM_131031.1	1.99
*rpl13α*	TCTGGAGGACTGTAAGAGGTATGC	AGACGCACAATCTTGAGAGCAG	148	NM_212784.1	1.94

Primers for *mmp9*, *muc2.2*, *β-def-1*, *fapb2*, and *fabp6 *were designed using AmplifX v1.4.0. Two reference genes, *β-actin* and ribosomal protein L13a (*rpl13α*), were used [10].
