# Supplementary material for: Lactoferrin Decreases the Intestinal Inflammation Triggered by a Soybean Meal-Based Diet in Zebrafish
Source: J Immunol Res. 2016 May 10;2016:1639720. doi: 10.1155/2016/1639720 (PMC4877474; doi:10.1155/2016/1639720)
Supplement: Supplementary file 1 — The supplementary Figure 1 shows the Sudan Black B staining in order to label leukocytes in the intestine of 9dpf larvae fed with the different diets. Total concordance between these result and those obtained from the immunohistochemistry analysis were found. [file 1639720.f1.docx]

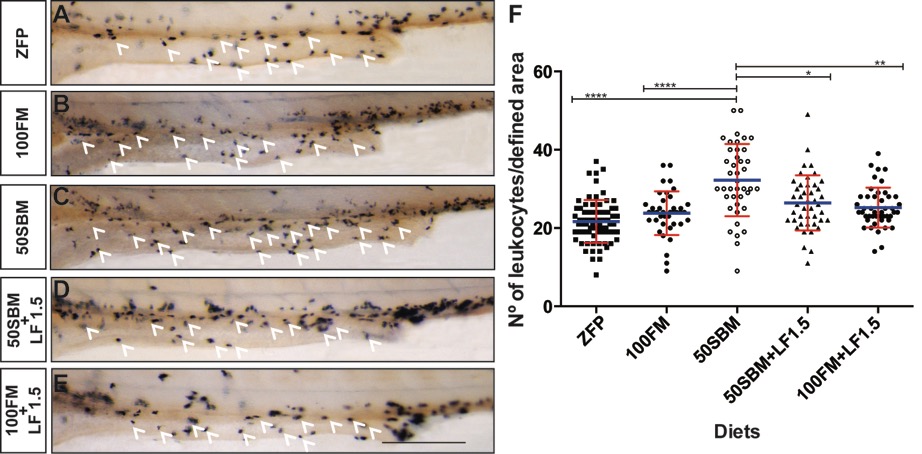


**Supplementary Figure 1. Effect of lactoferrin on the amount of leukocytes present in the intestine.** Sudan Black B staining was performed on 9dpf larvae, after four days of feeding with different diets (ZFP, 100FM, 50SBM, 50SBM+LF1.5, 100FM+LF1.5). (A-E) Lateral view of intestines, white arrowhead indicates leukocytes. (F) The experiments were conducted with at least 28 larvae per treatment in three different assays. Statistical analysis was performed by comparing data sets with the 50SBM diet through one-way ANOVA.The graph is a representation of three different results. *P < 0.05; **P < 0.01; ****P < 0.0001. Bar scale = 200 μm.
